# Motion changes response balance between ON and OFF visual pathways

**DOI:** 10.1038/s42003-018-0066-y

**Published:** 2018-06-07

**Authors:** Gloria Luo-Li, Reece Mazade, Qasim Zaidi, Jose-Manuel Alonso, Alan W. Freeman

**Affiliations:** 1 Sydney Medical School, The University of Sydney, Lidcombe, NSW 1825 Australia; 2 Graduate Center for Vision Research, College of Optometry, State University of New York, 33 West 42nd Street, New York, NY 10036 USA

## Abstract

Humans are faster at detecting dark than light stationary stimuli, a temporal difference that originates early in the visual pathway. Here we show that this difference reverses when stimuli move, making detection faster for moving lights than darks. Human subjects judged the direction of moving edges and bars, and made faster and more accurate responses for light than for dark stimuli. This light/dark asymmetry is greatest at low speeds and disappears at high speeds. In parallel experiments, we recorded responses in the cat visual cortex for moving bars and again find that responses are faster for light bars than for dark bars moving at low speeds. We show that differences in the luminance-response function between ON and OFF pathways can reproduce these findings, and may explain why ON pathways are used for slow-motion image stabilization in many species.

## Abstract (web summary)

Luo-Li et al. show that humans and cat cortical neurons respond faster to slowly moving light stimuli than to dark stimuli, contrary to previous findings for responses to stationary stimuli. The results may explain why ON visual pathways are used for slow-motion image stabilization in many species.

## Introduction

The visual system is organized in parallel ON and OFF pathways that process light and dark targets in visual scenes. ON and OFF pathways are present in many species, from flies^1^ to humans^2^. These pathways originate at the first synapse of the visual pathway in mammals and the second synapse in flies. The kinetics of this synapse are slower for ON than OFF pathways and, consistently, humans are also slower and less accurate at detecting lights than darks^3–5^. However, while the OFF pathway has better temporal resolution than the ON pathway^6,7^, slow-motion image stabilization is strongly dominated by the ON pathway^8^ through an ON directional selective cell^9,10^ that is among the best preserved retinal ganglion cells in mammals^11^. Slow-motion image stabilization is needed in almost all vertebrates to keep a steady image on the retina during self-movement, which is important to maximize visual acuity^12^. It’s unclear why the ON pathway dominates slow-motion image stabilization. Our results provide a possible explanation by demonstrating that the ON pathway is faster and more sensitive at processing slow motion compared to the OFF pathway.

## Results

### Psychophysics

**Fig. 1:**
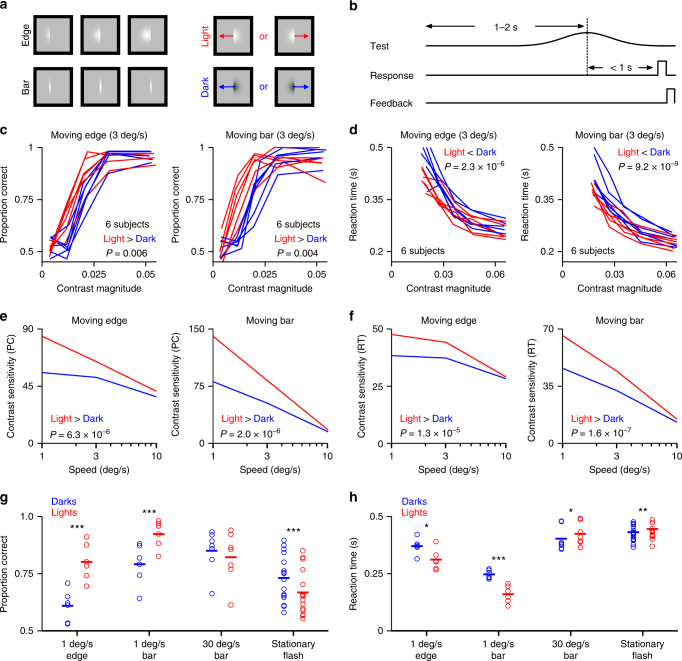
Humans see low speeds better with lights than darks. **a**, **b** Subjects detected the motion direction of dark/light drifting stimuli (edges/bars) presented at different contrasts. **c** Psychometric functions for accuracy obtained with light (red) or dark (blue) edges or bars. An analysis of variance showed that accuracy was better for light than for dark stimuli: *F*(1, 49) = 8.2 and 9.3 for edges and bars, respectively, where the factors were subject, contrast polarity, and powers of contrast magnitude from 1 to 4. *P*-values are shown on the graphs. **d** Same as (**c**) but for reaction time. Reactions were faster for light stimuli than for dark: *F*(1, 51) = 28 and 47 for edges and bars, respectively, with factors subject, contrast polarity, contrast magnitude and its square. **e** Contrast sensitivity was calculated by pooling psychometric functions across subjects (Supplementary Fig. 1a, c), finding the contrast at which proportion correct (PC) was 0.75, and taking the reciprocal of this contrast. Three stimulus speeds are shown. **f** Similarly, contrast sensitivity for reaction times (RT) was obtained from the contrast at which the pooled times (Supplementary Figure 1b, d) were halfway between their highest and lowest values. Contrast sensitivity for light stimuli is significantly greater than that for dark: *F*(1, 26) = 32, 37, 29, and 50 for the four graphs left to right, with factors subject, contrast polarity, speed and its square, and contrast polarity × speed. **g** Pair comparisons in the detection of light and dark stimuli presented at different speeds. Data for the stationary flash come from a reanalysis of results in Luo-Li et al.^4^. **h** Same as (**g**) for reaction time. Stars in (**g**) and (**h**) show significant values calculated with paired t-tests: *p < 0.05, **p < 0.01, ***p < 0.001

To quantify the speed at which humans perceive moving objects in psychophysical experiments, adult human subjects viewed edges or bars moving through a Gaussian window (Fig. 1a). On each trial the stimulus was lighter or darker than the background, with equal probability, and moved left or right, again with equal probability. The subject was asked to indicate the perceived direction of motion (Fig. 1a, b). Surprisingly, all six subjects were more accurate (Fig. 1c, *p* < = 0.006, ANOVA test) and faster (Fig. 1d, *p* < 0.001, ANOVA test) at detecting light than dark motion. This result was consistent across subjects and could be reproduced with both moving edges and bars (Fig. 1c, d, Supplementary Fig. 1a-d). The finding that humans are not always faster and more accurate at detecting dark than light stimuli is in contrast to what was previously thought^3,4^.

The difference in sensitivity between lights and darks was most pronounced at low speeds and was reduced as the speed increased (Fig. 1e, f, ANOVA tests, *p* < 0.001). The difference was reversed at high speeds (Supplementary Fig. 1e-f). On average, human subjects more accurately detected the slow motion of light than they did dark stimuli (Fig. 1g, 1 deg s^−1^ edge: 80 vs. 61%, 1 deg s^−1^ bar: 92 vs. 79%, *p* < 0.001, paired *t*-tests). Detection was also faster for lights than darks (Fig. 1h, 1 deg s^−1^ edge: 312 vs. 371 ms, *p* = 0.017, 1 deg s^−1^ bar: 161 vs. 248 ms, *p* < 0.001, paired *t*-tests). Conversely, when the stimuli were stationary and flashed (the fastest possible entrance of the stimulus in the receptive field), the detection was more accurate and faster for darks compared to lights (Fig. 1g, 73 vs. 67%, *p* < 0.001; Fig. 1h, 432 vs. 446 ms, *p* = 0.007, paired *t*-tests). Importantly, these differences were not due to a trade-off between accuracy and reaction time since the motion direction of lights was reported with both higher accuracy (Fig. 1c, e) and faster reaction time (Fig. 1d, f). From these experiments, we conclude that humans see slow motion better for lights than darks.

### Electrophysiology

**Fig. 2:**
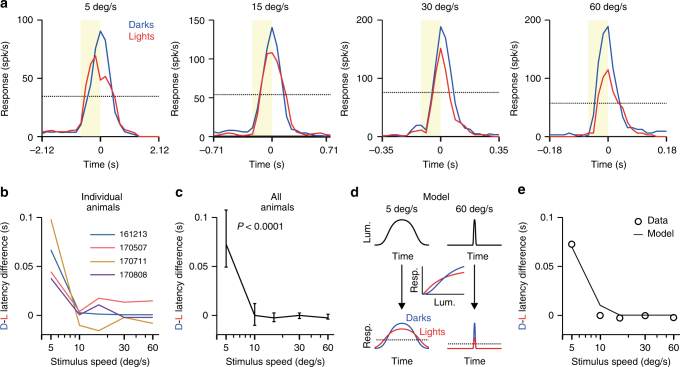
Different luminance-response functions between ON and OFF visual pathways explain the speed switch between lights and darks. **a** Cortical responses to light (red) and dark (blue) bars moving at different speeds. Notice the different time scales. The dotted line indicates the response level (half-amplitude for light bar) at which latency was measured. **b** Response latency differences between darks (D) and lights (L) measured in different animals. **c** Average difference in response latency. Error bars show 95% confidence intervals. **d** Latency differences explained with a model that uses different luminance-response functions for lights and darks (middle) to transform the stimulus (top) into peristimulus time histograms (dotted line is static threshold). **e** The model reproduces the data illustrated in (**c**)

To investigate the neuronal mechanisms underlying the low-speed advantage for lights, we recorded responses to light and dark bars swept across the receptive fields of neurons in cat primary visual cortex. As with human reaction times, cortical responses were faster for light than dark bars at low speeds but the difference was reduced when the bar speed increased. This trend could be demonstrated in recordings from single cortical sites (Fig. 2a), the average cortical response of individual animals (Fig. 2b), and the average cortical response across animals (Fig. 2c, *p* < 0.0001, bootstrap confidence interval). As expected from the strong OFF dominance of the visual cortex^13,14^, cortical responses were stronger for dark than light stimuli (Fig. 2a). However, when the stimulus speed was reduced, the responses to lights became faster than the stronger responses to darks (Fig. 2a, 5 deg s^−1^; see Supplementary Fig. 2a,b for additional comparisons of response strength and latency between lights and darks). The response latency differences between slowly-moving darks and lights were an order of magnitude larger than any possible latency artifacts due to the display monitor (Supplementary Fig. 3).

### Modelling

The different speed sensitivity to lights and darks could result from differences in the luminance-response functions between ON and OFF visual pathways^15–17^. To test this possible mechanism, we passed a Gaussian function through different ON and OFF luminance-response functions. Due to higher contrast sensitivity and response saturation for ON than OFF, this simulation made the Gaussian response broader and reach threshold faster for light than dark stimuli (Fig. 2d, 5 deg s^−1^), a difference that decreased with increasing stimulus speed (Fig. 2d, 60 deg s^−1^). At the highest speeds, the differences in response amplitude become more pronounced than the differences in response width and responses become faster to darks. This simple simulation reproduced the differences in response latency between lights and darks measured in the cat visual cortex (Fig. 2e).

## Discussion

In summary, our results demonstrate a functional advantage of the ON pathway in processing slow motion in humans and carnivores. The ON pathway is also more effective at processing slow motion in flies^18^, and zebrafish cannot correctly stabilize slow motion when the ON pathway is inactivated^19^. Therefore, the different response to speed between lights and darks that we demonstrate in humans and carnivores could be general across animals. The ON pathway has higher luminance sensitivity than the OFF pathway and its slower kinetics allows a more effective temporal integration of low-contrast edges during slow motion. The general properties of the ON pathway in animal vision (high sensitivity, slow kinetics) may therefore also explain why it is the chosen pathway for image motion stabilization.

## Methods

### Human psychophysics

A total of three female and three male human subjects took part in the experiments. Their ages ranged from 21 to 28 and they all had normal, or corrected to normal, vision in that acuity was 6/6 or better in each eye and stereo-threshold was 1 min or less. Subjects were unaware of the aims or results of the experiments. Stimuli were presented on a cathode-ray-tube monitor driven by an ATI Radeon HD 5770 video card. The card was controlled, and responses collected, with the Psychophysics Toolbox software extended by a low-level kernel driver^20^. Luminance was modulated with 10 bits per gun. Contrast is defined by (*L*_stim*−*_*L*_bg_)/*L*_*bg*_ where *L*_stim_ and *L*_bg_ are the stimulus luminance (maximum for lights, minimum for darks) and background luminance, respectively. Measurements of small luminance increments and decrements around the background level (obtained using a PR-650 photometer, Photo Research, Inc.) showed that measured contrast differed from the contrast setting by an average of 0.0018 (Supplementary Fig. 4a). Larger contrasts were linearized using a look-up table: fitting a straight line to the relationship between measured and set contrast yielded an adjusted *r*^2^ of 0.999, indicating a near-perfect fit (Supplementary Fig. 4b).

Most experiments used a monitor with a spatial resolution of 77 pixels deg^−1^, a 75 Hz video frame rate, a background luminance of 40 cd m^−2^, and a chromaticity of *x* = 0.34, *y* = 0.33. Supplementary Fig. 4c, d shows that light and dark moving bars had identical timing on this monitor, ruling out the possibility that reaction time differences were due to the monitor. For the experiments that required a stimulus speed of 30 deg s^−1^, the monitor characteristics were 46 pixels deg^−1^, 120 Hz, 32 cd m^−2^, and *x* = 0.31, *y* = 0.35. Subjects viewed the monitor from a distance of 1.14 m, used a chinrest to stabilize the head, and responded to stimuli by pressing one of two buttons on an RTBox^21^. Experiments were conducted in a quiet room in which the only substantial source of light was the monitor. Experimental procedures were approved by the University of Sydney Human Research Ethics Committee and informed consent was obtained from all subjects.

All stimuli were presented in a bordered area with inner diameter 2.5 deg × 2.5 deg. Borders were black and 0.25 deg wide. The upper part of Fig. 1a shows three frames from the moving edge stimulus. The image was multiplied by a Gaussian profile centered in the bordered area; the standard deviation was 0.3 deg. The stimulus was present until it was 0.25 deg from the border, or the subject responded, whichever was sooner. The moving bar, shown in the lower part of Fig. 1a, was windowed with the same Gaussian and was terminated in the same way as the edge. Stimulus speed was 1, 3, 10, or 30 deg s^−1^. Bar width was 0.1 deg except for the experiments using 30 deg s^−1^, in which case the width was 0.2 deg. On each trial the motion was leftward or rightward with equal probability. The subject’s task was to indicate motion direction.

Contrast on each trial was sampled from a Gaussian probability density with zero mean. Each stimulus therefore had equal probability of being lighter or darker than the background. The time course of a single trial is shown in Fig. 1b. Reaction time was measured relative to the midpoint time, that is, the time at which the edge or bar reached the center of the bordered area. The interval between the start of a trial and its midpoint time was sampled from a uniform probability density spanning the range 1 to 2 s. Subjects responded up to 1 s following the midpoint time or, if they failed to respond (usually because the stimulus was too faint), a medium-pitch auditory prompt was delivered and they were given another second in which to respond. Prompted responses contributed to the calculation of proportion correct but not to reaction time. This explains why the horizontal axis in Fig. 1d differs from that in 1c: low-contrast reaction times are missing and the bins are therefore centered on higher contrasts. In both unprompted and prompted cases, a low-pitch signal was sounded if the subject’s choice was incorrect. The next trial then started. Each run of trials lasted 60 s and subjects rested between runs if they wished.

Response differences between lights and darks were tested using analyses of variance. Factors were subject, contrast polarity, and powers of contrast magnitude from 1 to 4 (Fig. 1c and Supplementary Fig. 1a, c and e); subject, contrast polarity, contrast magnitude, and its square (Fig. 1d and Supplementary Fig. 1b, d and f); and subject, contrast polarity, speed and its square, and contrast polarity × speed (Fig. 1e, f).

### Animal physiology. Surgery and preparation

All surgical and recording procedures were performed in accordance with the US Department of Agriculture guidelines and were approved by the Institutional Animal Care and Use Committee (IACUC) at the State University of New York, State College of Optometry. Complete details of the surgical procedures have been described previously^13,22^. Briefly, adult male cats (Felis catus, *n* = 4) were tranquilized with an intramuscular injection of acepromazine (0.2 mg kg^−1^) and anesthetized with an intramuscular injection of ketamine (10 mg kg^−1^). Two intravenous catheters were inserted into each hind limb to administer continuous infusions of propofol (5–6 mg kg^−1^ h^−1^), sufentanil (10–20 ng kg^−1^ h^−1^), vecuronium bromide (0.2 mg kg^−1^ h^−1^), and saline (1–3 ml h^−1^). The animal was intubated, ventilated, and pupils dilated with 1% atropine sulfate with the nictitating membranes retracted with 2% neosynephrine. The eyes were fitted with contact lenses with a 3 mm pupil to focus on the monitor placed 57 cm in front of the animal. Throughout the surgery and recordings, the animal vital signs including temperature, electrocardiogram (EKG), expired CO_2_, electroencephalogram (EEG), pulse oximetry, and blood pressure were monitored and carefully maintained within normal physiological limits to ensure adequate anesthesia and ventilation.

### Electrophysiological recordings and data acquisition

Two 32-channel linear multielectrode arrays (0.1 mm inter-electrode distance, Neuronexus) were introduced horizontally and one 32-channel linear multielectrode array was introduced vertically in primary visual cortex to measure cortical multiunit activity; horizontal and vertical arrays were in opposite hemispheres. The horizontal multielectrode arrays were introduced into the cortex with <5° angle and centered in layer 4. The spike recordings were filtered between 250 Hz and 8 kHz, sampled at 40 kHz and collected by a computer running Omniplex (Plexon), as previously described.

### Visual stimuli and data analysis

Custom MATLAB code (Mathworks) with Psychtoolbox extensions was used to present visual stimuli on a 24-inch LCD gamma-corrected monitor (BenQ XL2420-B, 120 Hz, mean luminance: 120 cd m^−2^). The gamma correction was performed by measuring the input voltage and output luminance of the monitor (power function with a gamma exponent). A function in Psychtoolbox 3 was generated to correct for the gamma nonlinearity, thereby linearizing the relationship between contrast setting and displayed contrast.

The preferred direction/orientation of each cortical site was determined using moving light (240 cd m^−2^) and dark (0.27 cd m^−2^) bars (2.1 deg width) on a mid-gray background (120 cd m^−2^). The bars began at one side of the monitor and moved across the entire screen. The orientation of the moving bar was one of 16 possible values. For each dataset, the moving bars were presented at 6 speeds: 5, 10, 15, 17.3, 30, 60 deg s^−1^. Responses at 15 and 17.3 deg s^−1^ were similar and therefore combined; they are referred to as 16.15 deg s^−1^ (average of 15 and 17.3). Each dark and light oriented bar was presented four times, and peristimulus time histograms were compiled into 35 bins covering a stimulus sweep. To find the direction selectivity index for a recording site, we combined responses to light and dark for each motion direction, lightly smoothed impulse rate (Gaussian weighting with a standard deviation of 1 bin), and determined the maximum response, *R*, over all directions. The index was calculated as (*R*_pref_ *– R*_opp_)/*R*_pref_ where *R*_pref_ is the response for the direction in which response was maximal, and *R*_opp_ is the response in the opposite direction. Only cortical recording sites that showed clear direction selectivity were selected for analysis (direction selectivity index > 0.25).

We wished to average time courses over recording sites in order to look for light/dark asymmetries. Each site, however, receives its input from a slightly different location in the visual field and responses therefore peak at differing times. To compensate for these time differences, we fitted the model shown in Supplementary Fig. 2c and d (a Gaussian profile added to a linear function of time). The time course fitted with this model was then shifted laterally by subtracting the model’s peak time, and the shifted time course for the preferred direction was averaged over recording sites. Response latency was obtained by interpolating on the mean time course at the midpoint of the response with lower amplitude. The latency difference was calculated as the latency for the response to a dark bar less than for a light bar. The confidence intervals in Fig. 2c were calculated with a bootstrap procedure using 200 subsamples.

### Latency model

A simple model was constructed to replicate the light-dark latency differences measured in our recordings. A moving bar was first modeled as a Gaussian function to simulate the changes in luminance over time at the receptive field center, with narrower Gaussians simulating faster movement. Then, the Gaussian stimuli were passed through different ON and OFF luminance-response functions simulated as Naka-Rushton equations.

The parameters that varied in the model were the saturation of the luminance-response function and the spike threshold. The saturation of the luminance-response function was only allowed to vary within a narrow range that matched the physiological measurements obtained in cat visual cortex for light and dark stimuli presented on gray backgrounds^15,23^. The luminance-response saturation had to be always larger for lights than darks (as observed experimentally) and had to be constrained within a narrow physiological range (1 to 2.5 for the exponent and 0.3 to 0.5 for the *L*_50_ of the luminance-response function). The spike threshold was not needed to simulate the reduction (or elimination) of light-dark latency differences as velocity increased. However, using a spike threshold allowed a more precise match of the data and it was needed to simulate the sharp drop in latency differences at 10 deg s^−1^. The rest of the parameters used in the model were fixed including the stimulus time course (Gaussian width matching stimulus velocity used in experiments) and *R*_max_ (20% lower for light than dark stimuli as measured experimentally).

These parameters generated luminance-response functions with higher contrast sensitivity and saturation for ON than OFF pathways, as found in our experimental measurements from cats, monkeys, and humans^15,23^. In turn, the different luminance-response functions made Gaussian responses wider and weaker for light than dark stimuli. The differences in light-dark latency were calculated as the difference between the response time for lights and darks at half the maximum response to lights, as in the experimental measures. Below, we describe in more detail the equations for the different parts of the model and the parameters used in the simulation.

The inputs of the model were Gaussian functions (*stim*) whose width matched the duration of the stimulus at different speeds. The shape of the Naka-Rushton function was determined by the exponent (*n*) and the luminance that generated 50% of the maximum response (*L*_50_). The response magnitude was scaled by *R*_max_ (Eq. 1) and the spike threshold (*th*) was simulated as a power function.

1 $$R = \left( {R_{max}\frac{{stim^n}}{{L_{50}^n + stim^n}}} \right)^{th}$$

The *R*_max_, *L*_50_, and *n* were lower for ON than OFF pathways (*R*_max_: 80, *L*_50_: 0.4, *n*: 1.4 for ON; *R*_max_: 100, *L*_50_: 0.5, *n*: 2.3 for OFF) to make the saturation of the luminance-response function more pronounced for ON than OFF pathways, consistently with previous measurements in cats, monkeys, and humans^15,23^. The spike threshold values for each speed were arbitrarily chosen with the only requirement being that they had to increase with stimulus velocity (5 deg s^−1^: 1.0, 10 deg s^−1^: 2.0, 16.15 deg s^−1^: 2.5, 30 deg s^−1^: 3.0, 60 deg s^−1^: 3.5). Both the reduction in the dark-light difference-latency with velocity and the convergence of the light-dark difference at zero could be simulated without the spike threshold.

### Code availability

Computer code used to analyze data in this study is available from https://github.com/AlanFreeman/anaMotion.

### Data availability

The data that support the findings of this study are available from the corresponding author upon reasonable request.

## Electronic supplementary material

Supplementary Information
