# Supplementary material for: Motion changes response balance between ON and OFF visual pathways
Source: Commun Biol. 2018 Jun 7;1:60. doi: 10.1038/s42003-018-0066-y (PMC6123681; doi:10.1038/s42003-018-0066-y)
Supplement: Supplementary file 1 — Supplementary Information [file 42003_2018_66_MOESM1_ESM.pdf]

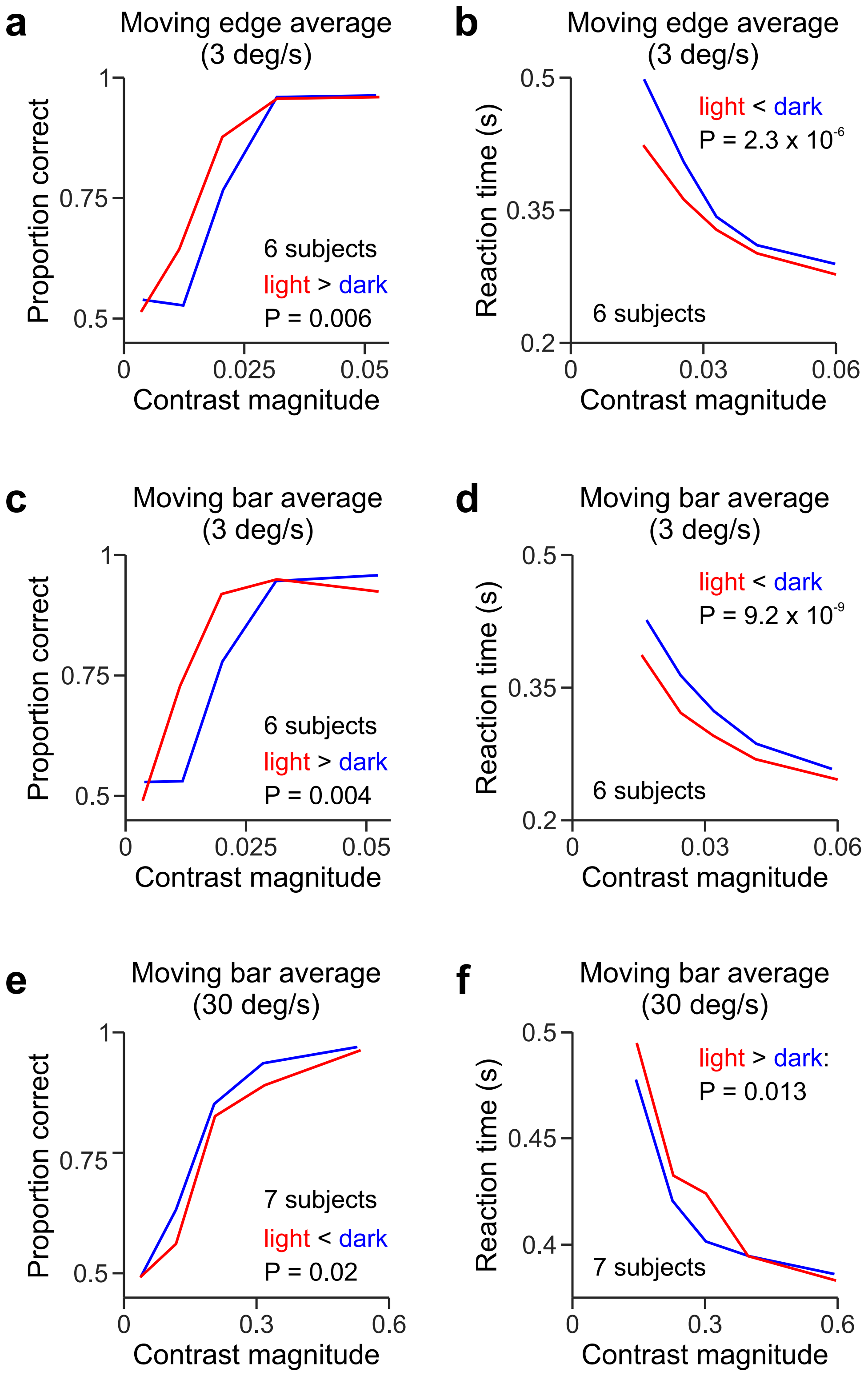


**Supplementary Figure 1. a – d.** Pooled psychometric functions, obtained with light (red) or dark (blue) stimuli moving at 3 deg/s, for the proportion of correct responses (a, c**)** and reaction time (b, d). The statistical tests are described in Figure 1. **e, f.** The stimulus speed here is 30 deg/s. Analyses of variance show that responses to darks are more accurate ($F(1, 58) = 5.7$ with factors subject, contrast polarity, and powers of contrast magnitude from 1 to 4) and faster ($F(1, 60) = 6.5$ with factors subject, contrast polarity, and contrast magnitude and its square) than for light stimuli. Note that the contrasts are ten-fold bigger at high speed because contrast sensitivity declines with speed (Figure 1e, f).

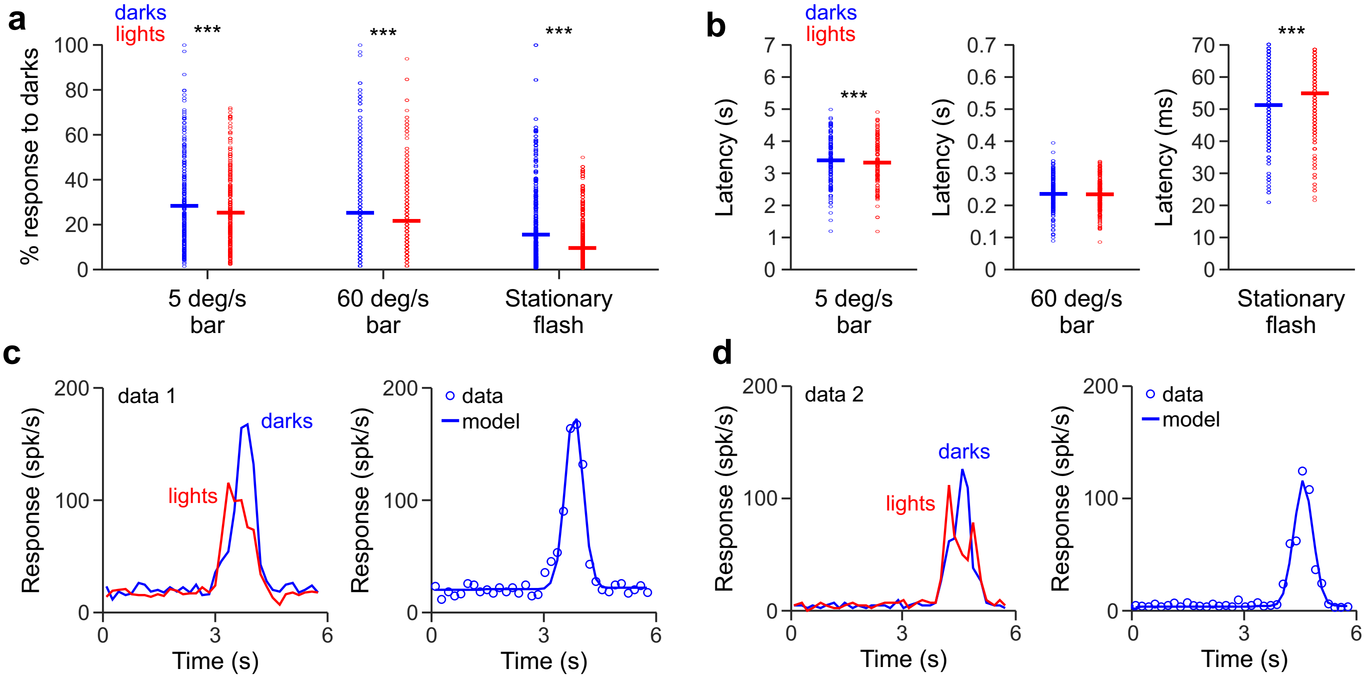


**Supplementary Figure 2. a.** Comparison of the strength of visual responses to light (red) and dark (blue) stimuli presented at different speeds. Response is measured as a percentage of the mean response to darks for each stimulus condition. Stars show significant values calculated with paired t-tests: *** p< 0.001. **b.** Same as a, but for response latency. **c.** The left side shows visual responses to light (red) and dark (blue) moving bars (5 deg/s), and the right side shows the response to a dark bar fitted with a model (Gaussian profile added to a linear function). This model was used to align the response time courses from different recording sites. **d.** Same as c, but for a second recording site.

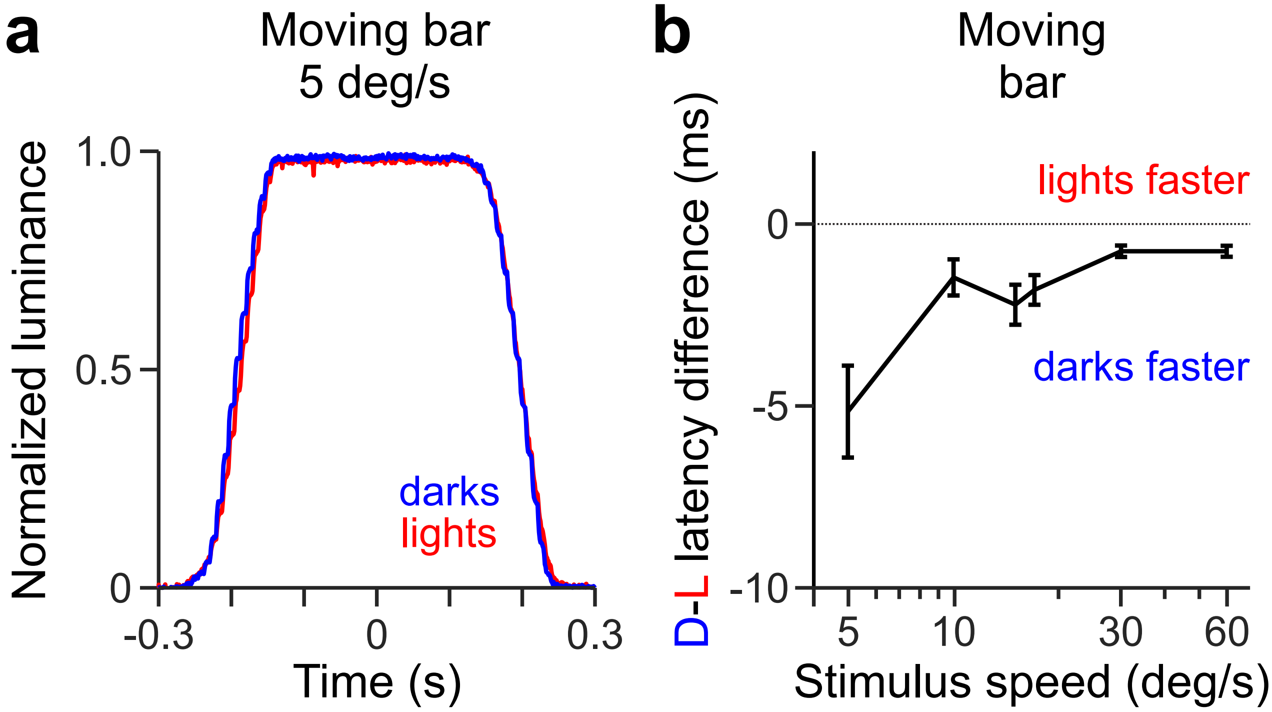


**Supplementary Figure 3.** Calibration of the LCD monitor used for the electrophysiological measures. **a.** Monitor responses to light (red) and dark (blue) stimuli measured with a photocell. **b.** Response latency differences between lights and darks measured with the photocell. Responses are faster to dark than light bars moved at slow speeds and the differences are reduced as the speed increases.

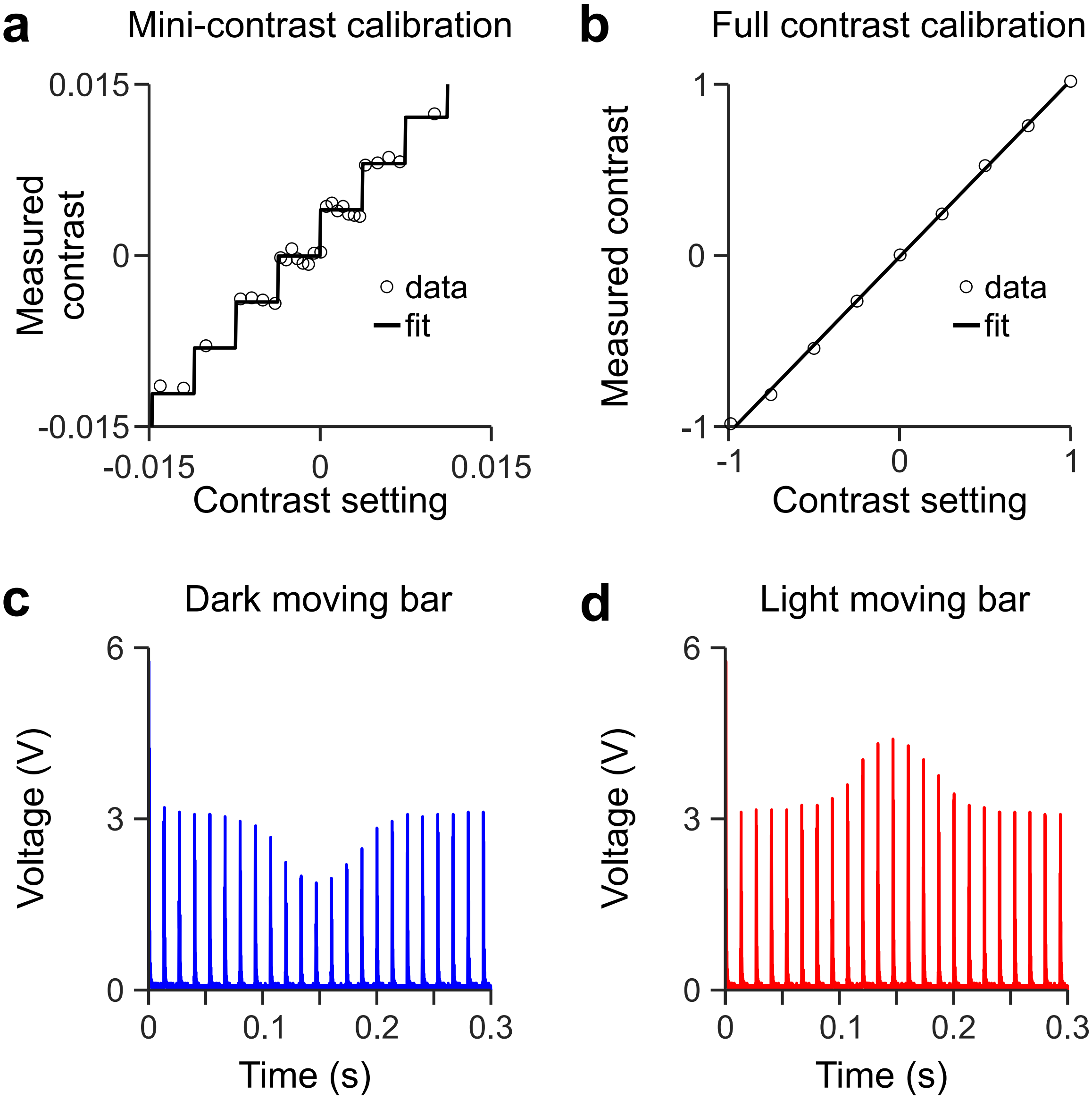


**Supplementary Figure 4.** Calibration of the cathode-ray-tube monitor used for psychophysical stimulation. **a.** Contrast was calculated by measuring luminances close to the background level. Measured contrast is a step function of contrast setting, reflecting the digital drive to the video card. **b.** Measured contrast is very close to contrast setting across the full contrast range. **c, d.** The vertical axis shows the output of a photosensor applied to the center of the monitor screen. Bars were moved at 3 deg/s across the screen: the trough of the dark bar response aligns with the peak of the light response, indicating that the psychophysical advantage for lights is not due to the monitor.
